# ERRATUM: Asthma and pregnancy

**DOI:** 10.1590/1806-9282.2023S123-ER

**Published:** 2025-11-03

In the manuscript: Carvalho-Pinto RM de, Cançado JED, Caetano LSB, Machado AS, Blanco DC, Garcia GF, et al. Asthma and pregnancy. Rev Assoc Med Bras. 2023;69(Suppl 1):e2023S123. https://doi.org/10.1590/1806-9282.2023S123

**Where it read:**

**Table 3:** Initial severity assessment of acute asthma in pregnancy.

Findings	Mild/moderate	Severe
Speaking in	Sentences or phrases	Words or unresponsive
Respiratory rate	18–19/min	>30/min
Heart rate	100–120 beats/min	>120 beats/min
Peak flow/FEV1 (%predicted)	50–75%	<50%
Pulse oximetry (room air)	90–95%	<90%

Reference: GINA^5^.

**It should read:**

**Table 3:** Initial severity assessment of acute asthma in pregnancy.

Findings	Mild/moderate	Severe
Talks	Phrases	Words
Respiratory rate	Increased	> 30/min
Heart rate	100-120 beats/min	> 120 beats/min
Peak flow	> 50%	< 50%
Pulse oximetry (room air)	90-95%	< 90%

Reference: GINA ^5^.

Roseli Nomura

*Scientifıc Editor*

